# Supplementary figures and images for: Using health information for community activism: A case study of the movement for change and social justice in South Africa
Source: PLOS Glob Public Health. 2022 Sep 15;2(9):e0000664. doi: 10.1371/journal.pgph.0000664 (PMC10022230; doi:10.1371/journal.pgph.0000664)

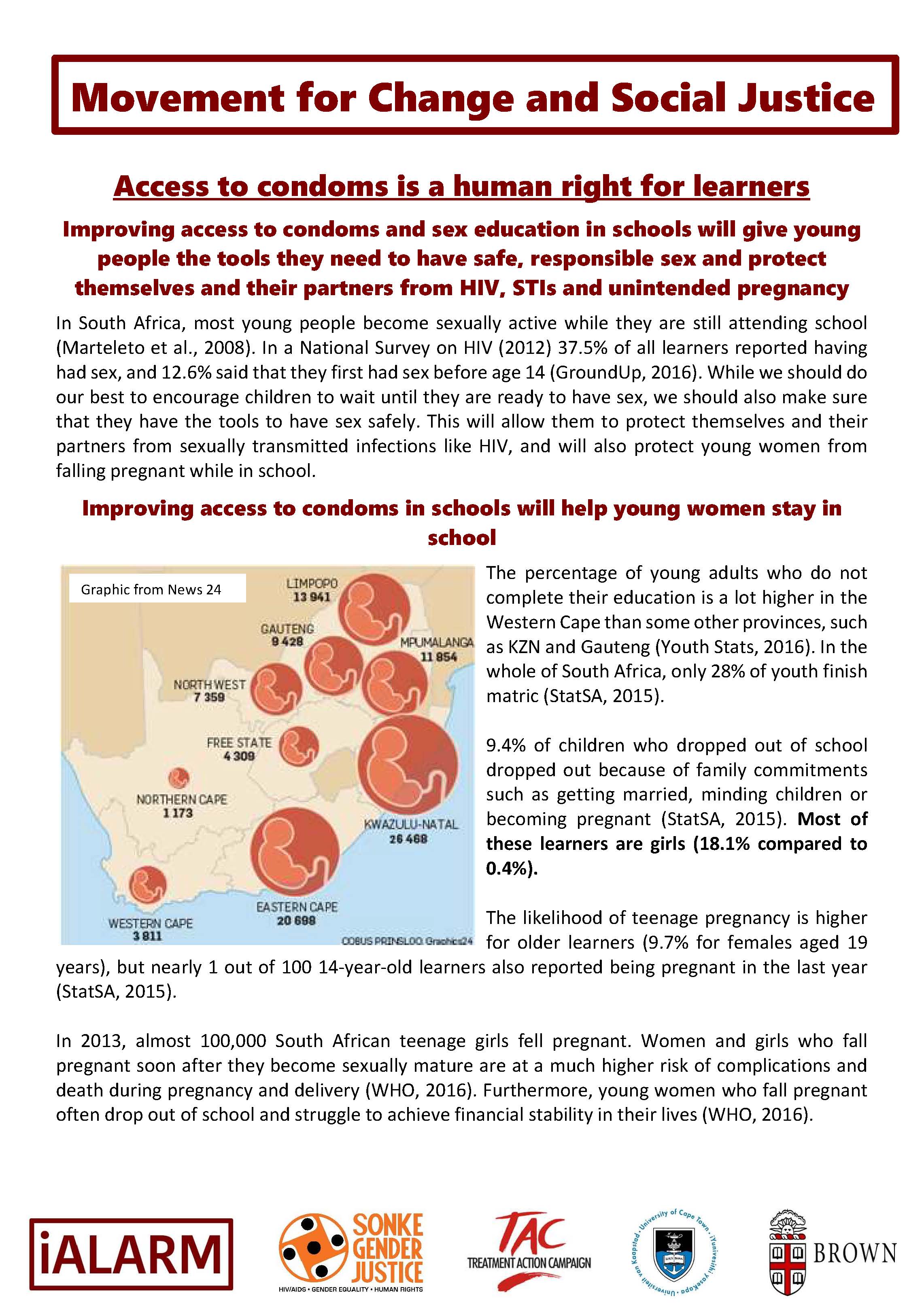
Appendix A: MCSJ Condoms in School Pamphlet


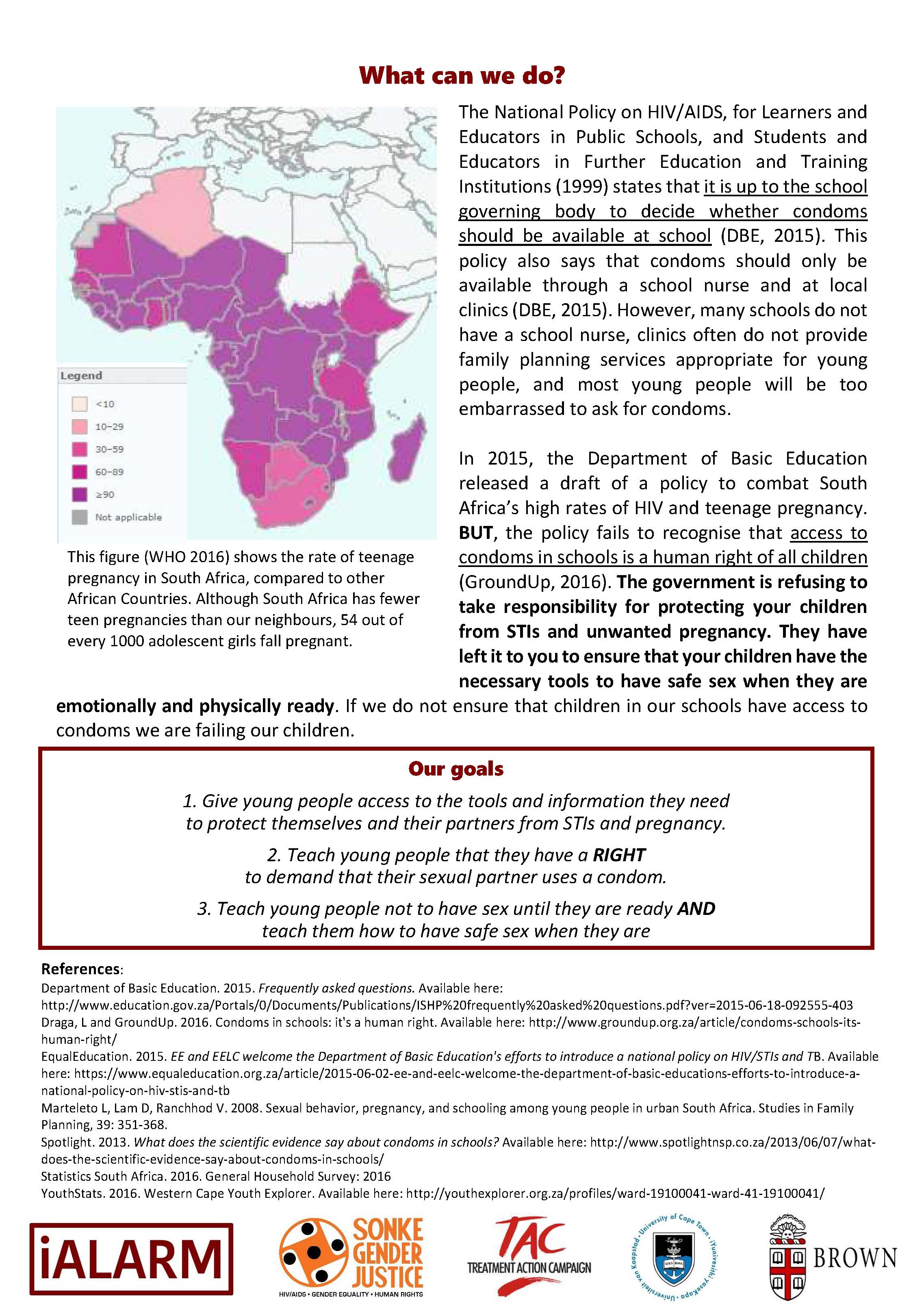

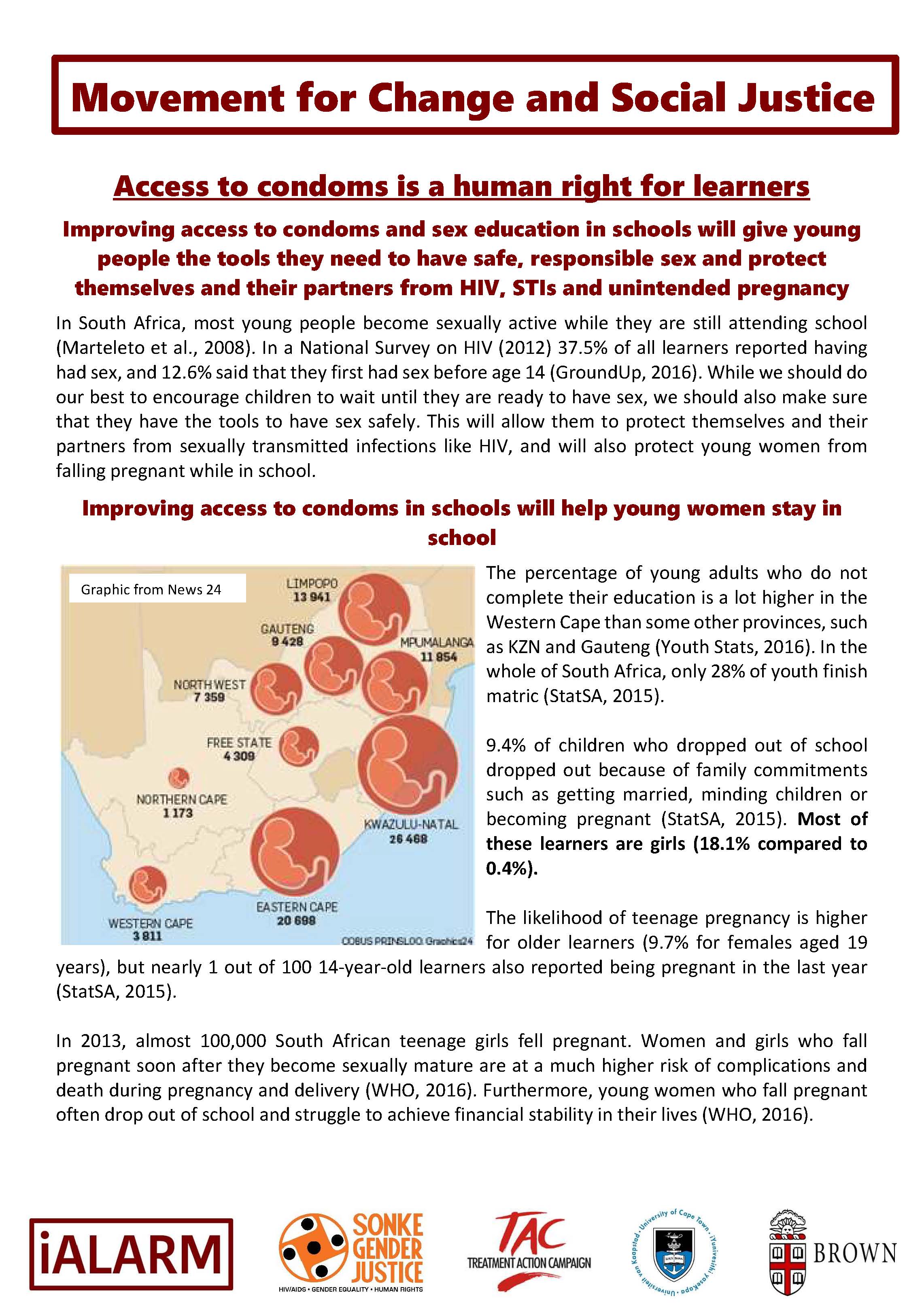

Supplement: S1 Text — (DOCX) [file pgph.0000664.s002.docx]
