# Supplementary material for: Using health information for community activism: A case study of the movement for change and social justice in South Africa
Source: PLOS Glob Public Health. 2022 Sep 15;2(9):e0000664. doi: 10.1371/journal.pgph.0000664 (PMC10022230; doi:10.1371/journal.pgph.0000664)
Supplement: Attachment — Submitted filename: Response to Reviewers 04042022.docx [file pgph.0000664.s003.docx]

Revisions in manuscript:

Reviewer 1:

| **Original transcript** | **Comment** | **Text revised transcript** | **Comment (if applicable)** | **Line number** |
| --- | --- | --- | --- | --- |
| Background |  |  |  |  |
| 1. | I would recommend a paragraph or so discussing the ethics of recording and sharing health data. | Additionally, South African data integrity policies are outdated which creates a continuous challenge to protect private patient information and in turn hinders the effective flow of health information across different levels of the health system (20, 21) | Thank you for the comment, I have added the following sentence in the background | 75 – 77 |
| 2. | I would recommend an initial breakdown of the different types of health data that you mention in your findings and a brief discussion on pros/cons of each type. You talk a lot about alternative types of health data in your findings/discussion but only briefly introduce informal, non-routine health data in the paragraph starting on line 74. | This formal and informal tacit knowledge is obtained through training, skill-building and work experience and considered to be the unwritten memory of an organization, which is hard to measure or quantify (21). Pamphlets, health education materials and patient experiences are other forms of non-routine health information (22). |  | 81-84 |
| 3. | Could you elaborate on the point of “over-produced and under-used” made in line 68? | In reality, however, health information is often over-produced by health actors, who routinely store patient data in routine management reports (RMR) and management reports, but largely under-used, due to fragmentation in the health system, shortages of human and financial resources and ill-defined information needs (11, 12) |  | 69-71 |
| Methods |  |  |  |  |
| 4. | I would personally suggest including the first section on MCSJ and Gugulethu in the background section. Set the scene earlier on. |  | Thank you so much for this comment. I have added MCSJs history to the background section. | 143 – 153 |
| 5. | In the paragraph starting on line 157 you detail information about Gugulethu. Could you elaborate on why the information you mentioned is relevant? What does this mean for your findings? Use this space to justify why you researched this initiative and why this area. What benefit will it bring to conduct research here? | There are three primary care facilities in the neighbourhood, which offer care for communicable and non-communicable diseases. Additionally several NGOs offer health education training and social support programmes. The field-site was deliberatelyGugulethu and the larger Klipfontein Sub-district were chosen, as iALARM’sresearch sites for the iALARM study. This project’s primary focus was to improveimproving the communication and coordination of services between the NY3 community health clinic in Gugulethu and the Men’s Wellness Centre, an initiative from Sonke Gender Justice (SGJ), located on the grounds of the clinic (14, 15, 42). | Thank you for your comment. I have added some more information in the methods section. | 170 – 176 |
| 6. | The methodology is missing some very significant information. What type of coding approach was used? How were the interviews recorded? Were they transcribed? You mention transcripts briefly in the ethical considerations, but don’t describe this earlier on. Make it precisely clear how you collected and analysed data. | All fieldnotes, observations, meeting minutes and interview transcriptions were written in English and stored on a password protected computer. This data was analysed in Nvivo, using an iterative thematic analysis approach used to ‘identify, analyse and report on patterns (themes) within the data’, whilst keeping the context in which the data was collected (11). This was a systematic and reflexive process which was largely conducted during data collection, using grounded theory (12). Firstly, broad emerging themes were verified across different fieldnotes and linked to the research questions. Later, categories and sub-themes were added and linked to the different examples described in Textbox 1-4. Then, MvP consulted with SC and CC for further refinement of the emerging themes. These included MCSJ’s capacity to create allies, to gain access to health information and the role of ‘safe space’ for effective health information sharing. Emerging themes were further crystallized after repeated discussion between the authors and feedback from key-informants in the project (13). | Thank you for your comment. I have completely rewritten the methodology section, and added more details, using the COREQ guidelines. I have added here the changes made in the data analysis section, but all other sections are also updated. | 208 - 220 |
| Findings |  |  |  |  |
| 8. | You introduce a lot of new concepts and make statements like “RMR is normally unavailable to the public” (line 245) that are not backed by references. These should be introduced and discussed in the introduction. How are these data collected, are they accessible, how could they help community groups, etc. | In reality, however, health information is often over-produced by health actors, who routinely store patient data in routine management reports (RMR) and management reports, but largely under-used, due to fragmentation in the health system, shortages of human and financial resources and ill-defined information needs (11, 12) |  | Line 69-71 |
| Discussion |  |  |  |  |
| 9. | The discussion is very good. Your idea of community groups engaging in a complex social process and portray them as actors engaging in multiple elements of health information sharing is very interesting. I feel you could highlight this point a little more strongly. |  | Thank you for this comment. After consideration with co-authors and reading comments from Reviewer #2, we think that we have emphasized the social process of data use and exchange sufficiently. |  |
| 10. | I would recommend a separate conclusion section to make your main arguments and recommendations clear to the users. | Conclusion:  This study explored the collection, use and exchange of health information among community activists to strengthen health service delivery in low-income settings in South Africa. By using unique strategies, MCSJ’s experience highlights that engagement with health information is a complex social process, and community stakeholders are valuable players within this process, as they are capable of linking health systems actors to patients. Therefore, when redesigning HISs to strengthen community based and patient-centred care, community groups such as MCSJ should be considered as critical partners. | Thank you for this comment. I have added the suggested conclusion section | 558 - 574 |
| Grammatical errors |  |  |  |  |
| 11. | As the rest of the article is written in British English, I have assumed to note the lack of “Oxford commas”.  Oxford comma missing on lines 30, 34, 48, 54, 59, 61, 63, 64, 66, 76, 85, 88, 92, 189, 239, 253, 402, 406, 424, 488, 493, 500 |  | Thank you for the comment, I have added the Oxford commas as per your suggestion. | Throughout |
| 12. | Line 33: comma after problematic unnecessary |  | Changed | Line 33 |
| 13. | Following sentences must be restructured as they are very difficult to follow: lines 38-40, 69-71, 177-179, 409-410 | Thank you so much for the comment. We have restructured several of the original sentences and deleted others. Here, we refer to the new version of the paper. | Through conducting in-depth interviews, participant observation and field-notes that were analysed using iterative thematic analysis, findings revealed that MCSJ effectively collected, used and exchanged health information to develop short-term health campaigns.  In reality, however, health information is often over-produced by health actors, who routinely store patient data in (RMR) reports and management reports. However, this information is under-used, due to fragmentation in the health system, shortages of human and financial resources and ill-defined information needs (11, 12). | Line 38-40  Line 67 – 71  178 - 181 |
| 14. | Line 422: Typo – “CHBISs” |  | Corrected in the text | Line 422 |

Reviewer 2:

| **Original transcript** | **Comment** | **Text revised transcript** | **Comment (if applicable)** | **Line number** |
| --- | --- | --- | --- | --- |
| Overall | This is clearly an important paper that outlines how a health activist group used health information to improve healthcare in a low-income area of Cape Town. I commend the authors for capturing the key strategies that were employed by the activist group to enact change. I have some comments that I hope would make the paper clearer and make the writing tighter. As a qualitative methodologist, I have focused on clarifying the methodology, ensuring the findings use quotes to demonstrate key themes that were derived from the interviews and following the reporting standards in checklists such as COREQ. |  | Thank you for your constructive comments. Please see COREQ checklist attached. We have also redrafted the methodology section.  Furthermore, we have added more quotes throughout the findings to strengthen our paper. |  |
| Background |  |  |  |  |
| 1. | For instance, it would be helpful, especially for those not familiar with the topic area, to explain what the authors mean by health information early in the background section. Health information is a broad term and it would be helpful to contextualise this to South Africa and provide an explanation of what it means in this article in relation to the WHO health system building blocks. |  | Thank you for the comment. We outline different types of health information in the second and third paragraph of the paper. |  |
| 2. | ‘This paper investigates…’ – The authors could build up to the need for this rather than have this upfront – more so because the background section ends with this important bit in lines 123 -131 anyway. Instead, authors could introduce a brief explanation of what they mean by health information at the end of the first para, so that it leads nicely into the second para that provides more detailed info on health information. |  | Thank you so much for your comment. After discussion between authors and feedback from reviewer 1, we decided to keep the structure of the original paper. |  |
| 3. | Line 82 – ‘some initiatives’ – where and by whom please? are these government initiatives? | Recently, health information researchers and development agencies based in low-middle income countries attempted to improve the access to information for non-health actors, mainly through the development of community health management information systems (CHMISs) or community-based health information systems (CBHIS). |  | Line 89 - 92 |
| 4. | Line 86 - are CBHISs the norm in all of Africa except in S.Africa as indicated from line 96 onwards? If there are variations across the rest of Africa, useful to avoid ‘In Africa’ and clarify this in line 86 (even saying ‘In some countries in Africa’ or similar would help). | Recently, health information researchers and development agencies based in low-middle income countries attempted to improve the access to information for non-health actors in Kenya and Zambia, mainly through the development of community health management information systems (CHMISs) or community-based health information systems (CBHIS). |  | Line 89-92 |
| 5. | Line 96 – are HFCs and clinic committees the same/similar? Apologies if I’ve got this wrong, but I’m assuming the key point made in this para is that while in some parts of Africa, there are CBHISs, in S.Africa this is not the case; so HFCs and clinic committees use other means to collect and use health information, although there isn’t much literature on this. That doesn’t come through clearly in this para, took a few readings - would really help to clarify. |  | Thank you for the comment. I agree with you and to avoid confusion, I have removed health facility committees (HFC) and only use clinic committees, as they are similar groups of people, but have different names in different settings. | Throughout |
| Methods | Kindly include a COREQ or similar checklist as an appendix (https://www.equator-network.org/reporting-guidelines-study-design/qualitative-research/). Including details in the manuscript to satisfy these criteria will also help address some of the key methodological queries raised. Please also strengthen the methods sections with appropriate references – there are none in submitted version. |  | Thank you for your comments. I have redrafted the methodology section and completed the COREQ checklist (sent as separate attached) | Methods |
| 6. | Lines 137 and 152 – repeats info but slightly different on short-term and long-term focus of MCSJ. | MCSJ is a community activism group established in 2016 in Gugulethu, MCSJ develops short- and long-term health campaigns to improve the quality of and access to health services in Gugulethu and neighbouring areas (Textbox 1-4). MCSJ was founded by Mandla Majola, an experienced HIV activist from Gugulethu. Through his activism background and role as community engagement manager in the iALARM study – a longitudinal study aiming to improve the testing and linkage of men to HIV services Mandla recognised the longstanding health issues in the community, including the high prevalence of HIV and non-communicable diseases, and lack of activist efforts (41, 42). Worried about the situation, he shared his concerns with other activists in Gugulethu, and collectively, they set-up the Movement for Change and Social Justice (MCSJ). MCSJ initially started with 15-20 committed activists, but has grown significantly over the past five years, establishing branches (offices) in other low-income neighbourhoods in Cape Town, including Nyanga, KTC, Kanana and Phillipi. |  | Line 143-153 |
| 7. | Line 184 – instead of extended period of time, specify for how long please. | All data was collected face-to-face. The ongoing relationship MvP established a close relationship with MCSJ during her between MvP and MCSJ that she established during her 18-month period of intensive fieldwork provided an opportunity for frequent conversations, reflections and discussions which were iterative in nature (5-7). MvP travelled to Gugulethu 2 or 3 times a week, depending on ongoing MCSJ activities. Findings from fieldnotes were discussed with key-informants to ensure data validity. Data was triangulated through informal conversations with key-informants, informed by queries and questions deriving from fieldnotes (8). Key areas of interests were MCSJs relationship with health providers and researchers, strategies used to access health information and role in the larger iALARM project. All interactions between the researcher and key-informants were in English and semi-structured interviewsconversations were audio-recorded and transcribed by MvP.. |  | 187 – 200 |
| 8. | Methods sections needs specific info as outlined in COREQ – for instance, were topic guides used for interviews, were interviews audio-recorded and transcribed, were interviews coded by more than one person, how many interviews/observations, etc. | Firstly, fieldnotes and interview transcripts were coded by MvP who identified emerging themes. These themes were verified broad emerging themes were verified across different fieldnotes and linked to the research questions. Later, categories and sub-themes were added, which became added and linked to the different examples described in Textbox 1-4. Then, MvP consulted with SC and CC for further refinement of the emerging themes. These included MCSJ’s capacity to create allies, to gain access to health information and the role of ‘safe spaces’ for effective health information sharing. Emerging themes were further crystallized after repeated discussion between the authors and feedback from key-informants in the project (48). | Thank you for your comment. In line with the COREQ guidelines, we restructured the methodology section. | Line 214 – 221 |
| 9. | Please consider rephrasing 'themes emerged' ‘emerging themes’ in manuscript in line with current thinking in qualitative research that these phrases does not take into account researchers' roles/is passive, as opposed to themes being 'identified', which is considered a more active process (please see Braun and Clarke's work, which elaborates on this). |  | Thank you for your comment. Please see comment and excerpt above. | Methods Section |
| Findings and discussion |  |  |  |  |
| 10. | Use of the term ‘Dentistry’ – sorry to be pedantic and this might be just down to differences in how we use these terms in different countries, so apologies if this is how it is used in S.Africa. As someone who used to be a dentist, to me, dentistry is usually used to mean dental medicine and not used to signify a place (where dental clinic is better) – like in line 233 and other places throughout manuscript. |  | Agreed, thank you for the comment. We have changed it throughout the text. | Throughout |
| 11. | I understand methods, such as observations were used, but given this is a qualitative research paper that used semi-structured interviews, one would expect to see more quotations – there is just one used. Please ensure findings that draw from interview data are adequately demonstrated with quotes (see COREQ). Similarly, it would be helpful to know which findings were drawn from which data sources so that it is clear to the reader that the findings are grounded in the data collected. |  | Thank you for your comment. We have reworked the methods based on the COREQ checklist (see attached).  We also added additional quotes to the findings as per your and reviewer 1’s suggestion. | Throughout |
| 12. | found it somewhat difficult to capture the key messages within each of the strategies – they could do with a really tight edit overall so that the most important findings within each are clear to the readers. It was also difficult to make out which aspects were from the data and which were the authors views based on their experiences or existing literature (note that the latter is important too, but perhaps findings is not the section for it) – e.g. lines 266-275 and similarly throughout the findings section. This paper would really benefit by keeping the findings tight and directly linked to the data from this study, with other aspects moved to discussion. |  | Thank you for the comments. We have significantly reworked the findings and tightened our arguments. The additional literature used in the findings is used to contextualize the findings.  Summaries of the findings sections are either removed or shortened to improve the flow and additional literature is only used to contextualise the findings. | Throughout |
| 13. | While it is important to reinforce some key messages, it would be important to not be repetitive – for instance, ‘social process rather than a technical exercise’ or similar is repeated three times in the discussion. | When designing these systems, the process of using and exchanging should be continuously considered. | Thank you for the comment. | 564 |
| 14. | Lines 460-461 – can’t see the ‘hunger for information’ mentioned in the findings. Ideal to not bring in new findings in the discussion, without mentioning them in the results first. |  | Thank you for the comment. We provided relevant references in the discussion | 529 |
| Ethical Issues |  |  |  |  |
| 15. | Line 198 – in what instances was verbal consent sought instead of written consent and why? | As data from key-informants was collected during meetings and group conversations, we were able to receive written consent, but obtain ongoing verbal consent from all participants. Key-informants were repeatedly informed that they could withdraw from the project at any time and were told that within group conversations, anonymity could not always be guaranteed. |  | 229-231 |
| 16. | Also, could you please indicate whether the people identified by name throughout the manuscript (activists, healthcare professionals, etc) have provided consent for the same. |  | Thank you for the comment. Please see revised text in response to comment 15. | 229-231 |
| 17. | Readers are likely to be curious about the informed consent aspects of the data collection activities of MCSJ, such as the quantitative data from patients in the queue for the dental clinic and the qualitative testimonials from ‘patients living with HIV’ (also, request to use people-first language instead of ‘HIV+ patients’ where possible). How was anonymity/confidentiality operationalised, given this information was then shared widely in local groups? Were any ethical issues in relation to this identified in the interviews, etc.? | “You need to get permission of the patient, and if he or she is afraid of victimisation we need to be keep them anonymous. But when they agree to give their ID number, exact names and addresses, it matters. To show, as an activist, that you are not falsifying and coming up with ghost people. If we want to keep the health system accountable, we need to be truthful and honest at all times.” | Thank you for this comment. In the findings, we added this additional quote. | 414-417 |
| Typos etc. |  |  |  |  |
| 18. | Line 31: Could you please amend to ‘However, many health system stakeholders, including community groups, are perpetually…’ | However, many health system stakeholders, including community groups are perpetually excluded from accessing and using health information,. |  | Line 31 |
| 19. | Line 123 – expand HIS please (it’s within other abbreviations before, but worth expanding separately |  | Thank you for the comment. I have added a separate list of acronyms/abbreviations |  |
| 20. | Line 170 – ‘who were recruited’ |  | Thank you for the comment. I have reworked the methodology section, so this sentence is no longer available |  |
| 21. | Line 172 – ‘when participating in observations of meetings’ |  | Thank you for the comment. I have reworked the methodology section, so this sentence is no longer available |  |
| 22. | Line 208 – ‘various creative’ |  | Thank you for the comment. I have reworked the methodology section, so this sentence is no longer available |  |
| 23. | Lines 443-444 – CBO or CTO? |  | It is CTO, Community Treatment Observatory (as explained in line 438) |  |
| 24. | Spotted a few more, but if paper is accepted these will be picked up in the proofreading process. For now, my view is that it is best to focus on tightening the paper so the messages are clear and ensure the methodological aspects are clear, with the inclusion of a COREQ checklist to help with that. Hope that helps – wishing you good luck with the paper, I enjoyed reading it |  |  |  |
