# Supplementary material for: Using health information for community activism: A case study of the movement for change and social justice in South Africa
Source: PLOS Glob Public Health. 2022 Sep 15;2(9):e0000664. doi: 10.1371/journal.pgph.0000664 (PMC10022230; doi:10.1371/journal.pgph.0000664)
Supplement: Attachment — Submitted filename: Comments Reviewers ROUND 2 ANSWERED 30052022 FINAL .docx [file pgph.0000664.s004.docx]

| Comment Reviewer 1 | Original text in the paper | Revised text | Response |
| --- | --- | --- | --- |
| 1. My key concerns are in relation to the ethical issues - the need for clearer justifications for the lack of written consent and the continuing use of participant names (possibly more problematic given the lack of written consent). |  |  | Thank you so much for the useful comments to this paper. Please see responses below. |
| a. The current section is somewhat confusing – says ‘we were able to receive written consent’ – was that meant to be ‘unable to receive written consent’? Assuming you meant 'unable', the justification provided is unclear to me - that it was because data was collected during meetings and group conversations; unsure why that prevents written consent if this was set out as a research project form the start. There are instances when written consent may not be possible, but it needs to be very clear why that was the case (what prevents written consent?) and if this was agreed upon with the ethics committee. This is a good piece of work that would hugely benefit form getting the ethics section really clear. | Ethical approval for the research was obtained from the Human Research Ethics Committee at the University of Cape Town (380/2017), the Western Cape Department of health (WCDoH) and the City of Cape Town (802/2014). All participants were informed about the objectives of the conducted research and ongoing iALARM study. As data from key-informants was collected during meetings and group conversations, we were able to receive written consent, but obtain ongoing verbal consent from all participants. Key-informants were repeatedly informed that they could withdraw from the project at any time and were told that within group conversations, anonymity could not always be guaranteed. | Ethical approval for the research was obtained from the Human Research Ethics Committee at the University of Cape Town (380/2017). The Western Cape Department of health (WCDoH) and the City of Cape Town (802/2014) gave access to conduct research in health facilities within the Klipfontein sub-district. As this research project was nested in the larger iALARM study and developed as an ongoing evaluation of the project, consent forms from key-informants in the iALARM study were obtained by iALARM researchers. All participants were informed about the objectives of the conducted research and linked iALARM study and at every point of contact, MvP explained her role as a researcher in the group. This research project was designed using an longitudinal ethnographic study design and aligned with disciplinary convention, MvP conducted research in larger meetings or public settings. Therefore, written consent could not be always be obtained from all participants (46, 50-52). While the use of pseudonyms is common in public health research, in ethnographic and anthropological disciplines, both the use of pseudonyms and real names are accepted, and both are considered respectful and appropriate, as long as written consent is obtained (46, 50-52). As our key-informants proudly presented the work they were doing in the community, we chose to identify them by name, after discussion and receiving written consent. Names of other participants are anonymised. | Thank you so much for this comment. We have tried to address your concerns by rewriting and clarifying the text in the methods section. See revised text.  *Lines 212 - 226* |
| b. I had requested information on whether you have explicit content to use names of participants (Lee, Mandla, Phumzile, etc.) You have added this sentence as justification - ‘were told that within group conversations, anonymity could not always be guaranteed’, which seems problematic to me. Adding the names of participants is extremely unusual even in research where we have written consent (which is the norm). Given we don’t have that in this study, I am struggling with how the naming of individuals is ethically justifiable without written consent. |  |  | Thank you for your comment. As per response above 2a, I have kept in all the names of participants who gave written consent to be named in this paper, and have taken out the name of one person from which I did not receive written consent.  *Throughout the text* |
| 2. Methods a. Please follow standard norms in how participant quotes are labelled. They have to be numbered in some way, with an explanation for that (see COREQ 29; latter part of it has not been addressed). Using the month and year does not really help with working out if you have been able to use a wide range of quotes nor does it give us information about the participant’s profile. |  |  | Thank you for your comment. This is a relevant point.  Instead of giving participants unique ID numbers, we have identified all quoted of participants with their relevant role in project (MCSJ member, NGO representative, nurse etc.) and their respective place of work (Gugulethu, Gugulethu Community Health Centre etc.). Example: (*Facility manager, Gugulethu day clinic*)  *Throughout the text* |
| b. You had semi-structured interviews in the original, which is now replaced with informal conversations. But you later mention ‘interview transcripts’ in analysis without referring to ‘interviews’ in data collection; also abstract still has interviews in it – all very confusing unfortunately! Please be very clear about the methods used and ensure this is consistent in every part of the manuscript, including in COREQ (no. 17 for this needs to be addressed if interviews were conducted). |  | As a primary researcher, MvP collected data for this paper using a variety of ethnographic methods, which include participant observation, shadowing, informal conversations and semi-structured interviews.  Through participant observation, shadowing, informal conversations and semi-structured interviews that were analysed using iterative thematic analysis, findings revealed that MCSJ effectively collected, used and exchanged health information to develop short-term health campaigns. | Thank you for your comment. This was confusing. I have now added all relevant methods of ethnographic data collection in the abstract and the text.  *Lines: 178 – 180 in text and 38 – 40 in abstract* |
| c. COREQ – no. 22, please address. |  | All data was collected face-to-face. MvP established a close relationship with MCSJ during her 18-month period of intensive fieldwork provided an opportunity for frequent conversations, reflections and discussions which were iterative in nature (5-7). *LINES 181 – 183*  To navigate this space as an ‘outsider’ emerged in researcher the social movement, MvP had continuous conversations with SC and CC about her positionality and regularly shared her observations and concerns. MvP also travelled to Gugulethu 2 or 3 times a week for 18 months, allowing her enough time to reflect on her findings (10). *LINES 193 – 197* | Thank you so much for you comment. Although not stated explicitly within the paper, data saturation was reached due to the length that MvP spent in the field, 18 months. Although evaluation of the iALARM project was still ongoing, MvP felt satisfied with the collected data, due to the time period she spent conducting fieldwork and engaging with key-informants.  *Please see revised text* |
| d. I think my comment on themes emerged may not have been clear. Please avoid phrases ‘themes emerged’ or ‘emerging themes’ and replace with ‘themes identified’ or ‘generated themes’ or similar in three places in the data analysis sections (this is because current thinking is that when we say themes 'emerged', it sounds like a passive process independent of the researcher; whereas identified/generated take the role of the researcher into account and signify an active process). | Firstly, fieldnotes and interview transcripts were coded by MvP who identified emerging themes. These themes were verified themes were verified across different fieldnotes and linked to the research questions. Later, categories and sub-themes were added. Then, MvP consulted with SC and CC for further refinement of the emerging themes. These included MCSJ’s capacity to create allies, to gain access to health information and the role of ‘safe spaces’ for effective health information sharing. Emerging themes were further crystallized after repeated discussion between the authors and feedback from key-informants in the project (48). | This was a systematic and inductive process which was largely conducted during data collection, using grounded theory (48). Firstly, fieldnotes and interview transcripts were coded by MvP who identified themes. These themes were verified across different fieldnotes and linked to the research questions. Later, categories and sub-themes were added. Then, MvP consulted with SC and CC for further refinement of generated themes. These included MCSJ’s capacity to create allies, to gain access to health information and the role of ‘safe spaces’ for effective health information sharing. Generated themes were further crystallized after repeated discussion between the authors and feedback from key-informants in the project (49). | Thank you so much for the useful comments. We have changed ‘emerged’ to ‘verified’ or ‘identified’ within the data analysis section.  *Lines: 203 – 209* |
| 3. Data availability – says all uploaded as appendix, but cannot find the link. |  |  | Thank you so much for your comment. After consultation with A/Prof Christopher J. Colvin, co-author of this paper and Co-PI of the iALARM project, I confirmed we are not able to share any raw data. The original iALARM ethics agreement with UCT’s HREC, which was obtained in 2014, indicates that we would keep all qualitative research data confidential and would publish interpreted findings only. |
| A number of minor typos as a result of changes made need some attention – PLHIV and PLWH used? Spotted quite a few others, especially around the track changes (some edits have meant context may have been lost, so may need a really good read through by the authors to ensure all the pieces fit together); hopefully some of this will be picked up at proofreading stage if accepted. Good luck with this. |  |  | Thank you for your comments. We have done a thorough check of the revised manuscript. |
